# A novel interaction between kinase activities in regulation of cilia formation

**DOI:** 10.1186/s12860-017-0149-5

**Published:** 2017-11-15

**Authors:** Nicole DeVaul, Katerina Koloustroubis, Rong Wang, Ann O. Sperry

**Affiliations:** 1 Anatomy and Cell Biology, East Carolina University, Brody School of Medicine, Greenville, NC USA; 2 Laboratory of Biochemistry and Genetics, National Institute of Diabetics and Digestive and Kidney Diseases, National Institutes of Health, Bethesda, MD USA

**Keywords:** Cilia, AurA, Nek2, PP1, PPP1R42

## Abstract

**Background:**

The primary cilium is an extension of the cell membrane that encloses a microtubule-based axoneme. Primary cilia are essential for transmission of environmental cues that determine cell fate. Disruption of primary cilia function is the molecular basis of numerous developmental disorders. Despite their biological importance, the mechanisms governing their assembly and disassembly are just beginning to be understood. Cilia growth and disassembly are essential events when cells exit and reenter into the cell cycle. The kinases never in mitosis-kinase 2 (Nek2) and Aurora A (AurA) act to depolymerize cilia when cells reenter the cell cycle from G_0_.

**Results:**

Coexpression of either kinase with its kinase dead companion [AurA with kinase dead Nek2 (Nek2 KD) or Nek2 with kinase dead AurA (AurA KD)] had different effects on cilia depending on whether cilia are growing or shortening. AurA and Nek2 are individually able to shorten cilia when cilia are growing but both are required when cilia are being absorbed. The depolymerizing activity of each kinase is increased when coexpressed with the kinase dead version of the other kinase but only when cilia are assembling. Additionally, the two kinases act additively when cilia are assembling but not disassembling. Inhibition of AurA increases cilia number while inhibition of Nek2 significantly stimulates cilia length. The complex functional relationship between the two kinases reflects their physical interaction. Further, we identify a role for a PP1 binding protein, PPP1R42, in inhibiting Nek2 and increasing ciliation of ARPE-19 cells.

**Conclusion:**

We have uncovered a novel functional interaction between Nek2 and AurA that is dependent on the growth state of cilia. This differential interdependence reflects opposing regulation when cilia are growing or shortening. In addition to interaction between the kinases to regulate ciliation, the PP1 binding protein PPP1R42 directly inhibits Nek2 independent of PP1 indicating another level of regulation of this kinase. In summary, we demonstrate a complex interplay between Nek2 and AurA kinases in regulation of ciliation in ARPE-19 cells.

**Electronic supplementary material:**

The online version of this article (10.1186/s12860-017-0149-5) contains supplementary material, which is available to authorized users.

## Background

Primary cilia are microtubule-based organelles that protrude from the cell membrane to receive and transduce environmental signals. Interference with the formation and/or stability of cilia disrupts signaling pathways essential for normal development and maintenance of the differentiated state ([1–3]; as reviewed in [4, 5]). A diverse collection of developmental disorders, ciliopathies, stem directly from disruption of ciliary function and display a wide range of abnormalities from cystic kidney to obesity (as reviewed in [6, 7]). Virtually all cells form primary cilia, which are structurally analogous to flagella. Cilia assemble when cells exit the cell cycle. As cells reenter the cell cycle and begin to proliferate, cilia disassemble, the basal body detaches from the plasma membrane, and centrosomes duplicate to form the mitotic spindle. Although the inventory of proteins that constitute cilia is increasing, the mechanisms regulating their formation and disassembly are just beginning to be defined.

**Table 1:** Summary of percentage cilia and length for transfections

	Percentage cilia			Length, μm		
	Cycling	Assemb	Disassemb	Cycling	Assemb	Disassemb
Empty	1.7 ± 0.8	42.1 ± 3.7	12.6 ± 1.4	1.7 ± 0.4	4.1 ± 0.1	2.9 ± 0.1
AurA	0	26.5 ± 2.8	8.8 ± 0.9	0	4.1 ± 0.1	3.1 ± 0.2
Nek2	0.5 ± 0.5	27.8 ± 2.3	7.2 ± 1.9	0.9 ± 0.5	4.2 ± 0.1	3.0 ± 0.1
AurA/Nek2	ND	13.3 ± 1.0	15.5 ± 1.6	ND	7.2 ± 0.2	7.0 ± 0.3
AurAKD	ND	31.7 ± 1.8	13.6 ± 1.3	ND	4.5 ± 0.2	4.0 ± 0.1
Nek2KD	ND	32.2 ± 1.2	16.8 ± 1.8	ND	5.3 ± 0.2	3.2 ± 0.2
AurAKD/Nek2KD	ND	34.5 ± 2.7	25.6 ± 1.5	ND	14.0 ± 0.8	10.1 ± 0.3
AurA/Nek2KD	ND	12 ± 1.3	18 ± 2.0	ND	3.9 ± 0.1	3.3 ± 0.1
AurAKD/Nek2	ND	11 ± 1.0	19.6 ± 2.0	ND	3.4 ± 0.2	3.0 ± 0.1
R42	9.1 ± 1.3	47.2 ± 2.9	22.3 ± 3.0	4.2 ± 0.3	4.3 ± 0.1	3.4 ± 0.2
R42/AurA	1 ± 0.4	24.8 ± 2.1	7.2 ± 1.0	2.6 ± 0.7	4.3 ± 0.1	2.7 ± 0.1
R42/Nek2	0.8 ± 0.4	18.4 ± 2.6	12.5 ± 2.6	2.2 ± 0.8	4.0 ± 0.1	3.0 ± 0.1
OT siRNA	4.8 ± 1.3	46 ± 4.6	26.3 ± 8.6	2.6 ± 0.2	4.0 ± 0.1	2.9 ± 0.1
R42 siRNA	3.4 ± 0.7	41 ± 2.2	24 ± 4.0	2.7 ± 0.2	3.6 ± 0.1	3.4 ± 0.2
Empty-MLN	ND	26.4 ± 3.1	13.2 ± 1.3	ND	6.4 ± 0.4	5.3 ± 0.4
Empty + MLN	ND	33.4 ± 1.7	18.3 ± 3.3	ND	6.8 ± 0.4	4.8 ± 0.4
Nek2-MLN	ND	25.2 ± 2.3	10.6 ± 1.5	ND	5.9 ± 0.4	4.8 ± 0.4
Nek2 + MLN	ND	36.2 ± 2.0	17.5 ± 1.4	ND	6.3 ± 0.3	4.8 ± 0.4
Empty-rac	ND	46 ± 3.7	17.6 ± 2.0	ND	4.6 ± 4.7	5.3 ± 0.6
Empty + rac	ND	43 ± 5.8	27.7 ± 2.3	ND	10.8 ± 1.2	6.4 ± 0.6
AurA-rac	ND	33 ± 4.4	14.3 ± 2.3	ND	5.3 ± 0.7	3.8 ± 0.3
AurA + rac	ND	48.4 ± 5.6	28 ± 5.0 l	ND	7.4 ± 0.9	11.8 ± 1.3

The mean percentage of cilia number and length ± SEM is shown for each transfection

Cilia display regulated growth and retraction when entering and exiting the cell cycle. Cilia grow as cells enter G_0_ and are absorbed prior to reentry into the cell cycle. Two important regulators of cilia absorption are the kinases Nek2 (NIMA related kinase 2) and AurA (Aurora A). Nek2 is a serine/threonine kinase that is localized to the distal portion of the mother centriole and functions in both cilia shortening and centrosome duplication [8, 9]. Nek2 controls cilia disassembly; depletion of Nek2 causes an increase in the number of ciliated cells [8]. Nek2 has been linked directly to disruption of left-right asymmetry, a biological consequence of cilia dysfunction [10]. Nek2 also regulates intraflagellar transport (IFT) through phosphorylation of the kinesin KIF24 to stimulate cilia depolymerization [11]. Additionally, Nek2 induces centrosome separation prior to cell division. Overexpression of active Nek2 causes premature splitting of centrosomes [12] due to phosphorylation and destabilization of centrosomal linker proteins [9].

AurA, another serine/threonine kinase, is essential for maintenance of cilia length and cilia retraction prior to cell cycle reentry in diverse organisms [13, 14] as well as centrosome maturation, duplication, and spindle assembly (as reviewed by [15]). Pioneering work in *Chlamydomonas reinhardtii* provided the first indication that AurA regulates the length of the flagellum of this biflagellate alga [16, 17]. AurA is localized to and activated at the basal body of cilia when cilia disassemble. Overexpression of AurA in ciliated mammalian cells induces cilia disassembly through activation of a tubulin deacetylase [13]. Like Nek2, AurA participates in preparation of centrosomes for cell division (reviewed in [18–20]).

PP1, a serine/threonine phosphatase, is a common regulator of both kinases in control of centrosome separation prior to spindle formation at mitosis; however, its role in cilia biogenesis has not been investigated [19–22]. PP1 activity is itself regulated by both positive and negative regulatory subunits. The negative regulator PPP1R2 (I2) inhibits PP1 activity in both centrosome separation and cilia acetylation and stabilization [19, 23]. We have previously identified a PP1 binding protein, PPP1R42 that is involved in centrosome separation [24]; however, its role in ciliation is not known.

Our study provides evidence that Nek2 and AurA interact differentially depending on cilia growth status. We demonstrate that Nek2 and AurA interact on several levels. They appear to share positive and negative factors to enhance or inhibit depolymerization activity when cilia are disassembling or assembling, respectively. Nek2 and AurA act independently when cilia are growing but both are required to depolymerize cilia. Furthermore, we demonstrate that these two kinases act additively to depolymerize cilia when cilia are growing and are independently involved in cilia number and length. These findings represent a novel functional interaction between two kinases involved in cilia disassembly. In addition, we identify inhibition of Nek2 by PPP1R42, a PP1 binding protein, which is independent of PP1.

## Results

### Requirement for kinase activity is dependent on cilia growth state

**Fig. 1:**
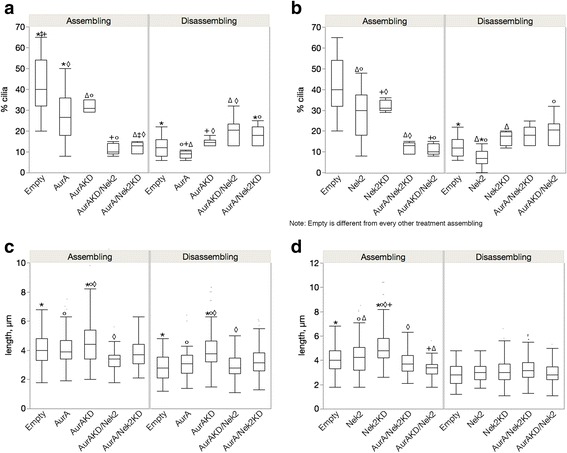
The effect of active kinase and dominant negative enzymes alone or in combination. ARPE-19 cells were transfected individually with AurA, AurAKD (kinase dead dominant negative), Nek2 and Nek2KD (kinase dead dominant negative), AurKD/Nek2, AurA/Nek2KD. Cilia number (**a** and **b**) and length (**c** and **d**) were measured for the AurA single and cotransfectants (**a** and **c**) and the Nek2 single and double transfectants (**b** and **d**). Measurements were made when cells were serum starved for 48 h (assembling cilia, left panels) and refed serum for 24 h (disassembling cilia, right panels). 300 cells were counted. Significant differences are indicated by like symbols; *p* ≤ 0.05. Empty vector is significantly different from all other transfectants when cilia are assembling. The means for cilia number and length ± SEM are shown in Table 1

We investigated the interaction between AurA and Nek2 by overexpressing the kinases and their kinase dead counterparts either alone or in combination in cells either growing cilia after serum starvation or absorbing cilia after reintroduction of serum (Fig. 1). The kinase dead versions of Nek2 and AurA have been shown to localize to the centrosome and to have a dominant negative effect on endogenous kinase function by sequestering substrates and upstream regulators of the kinases (Dr. Andrew Fry, personal communication and [12, 25, 26]). Expressed protein is maintained throughout the time course of treatment (Additional file 1: Figure S1) with a transfection efficiency of 90% on average (Additional file 2: Figure S2) and cells show little toxicity after transfection. These experiments examine a time window between formation of cilia after cell division and before the approximate onset of the next division. The effect of experimental manipulation on cilia in cell populations has precedent and results of such studies have expanded our knowledge of cilia biology [13, 27–29].

We first compared cilia number and length in cells expressing either kinase active or kinase dead versions of AurA and Nek2 in cells assembling or disassembling cilia to determine if the effect on cilia depolymerization differed depending on cilia growth status (Fig. 1, compare Empty (control) vs AurA, and Empty (control) vs Nek2). Previous studies have shown that AurA and Nek2 induce cilia depolymerization [11, 13]; therefore, we expected that cells transfected with the active kinases would be less ciliated than control. This was true for cells assembling cilia (Fig. 1A, left panel, AurA vs control, *p* = 0.008; Fig. 1B left panel, Nek2 vs control, *p* = 0.0009) but not for cells disassembling cilia. Only Nek2 transfected cells were significantly less ciliated than control (Fig. 1B, right panel, *p* = 0.03). There was no significant difference in cilia length between active kinase and control (Fig. 1C, AurA vs control; Fig. 1D, Nek2 vs control). The stronger effect of the active kinase compared to control in cells assembling cilia may reflect a shift in the balance from assembling to disassembling catalyzed by the active kinases. The active kinases are not as effective at further depolymerizing cilia when cilia are already disassembling (Fig. 1A and B, right panels).

We expected that cells transfected with the kinase dead enzymes would have more and longer cilia compared to control and wild-type (compare Fig. 1 control and AurA vs AurAKD, and control and Nek2 vs Nek2KD). However, the kinase dead enzymes of AurA and Nek2 did not increase cilia number compared to control either when cilia are assembling or disassembling which may suggest that ciliation is at a maximum and the kinase dead version cannot further stimulate cilia formation above control levels (Fig. 1A and B). The KD was significantly different from the active kinase only when cilia were disassembling (Fig. 1A, right panel, AurA vs AurAKD, *p* = 0.047; Fig. 1B, right panel Nek2 vs. Nek2KD, *p* = 0.004) suggesting that the observed destabilization of cilia by the active kinase is dependent on its kinase activity when cilia are disassembling but not assembling.

We also measured cilia length in cells transfected with the kinase dead enzyme and compared the values to wild-type and control cells (Fig. 1C and D). Cilia were significantly longer in cells transfected with the kinase dead compared to control when cilia were assembling (Fig. 1C, left panel, AurAKD vs control, *p* = 0.01; Fig. 1D left panel, Nek2KD vs control, *p* < 0.0001) and for AurKD when cilia were disassembling (Fig. 1C, right panel, AurAKD vs control, *p* < 0.0001). Cilia were also significantly longer when the KD was compared to the wild-type enzyme in cells assembling (Fig. 1C, left panel, AurA vs AKD, *p* = 0.02; Fig. 1D, left panel, Nek2 vs Nek2KD, *p* < 0.0001) or disassembling cilia (Fig. 1C, right panel, AurA vs AKD, *p* = 0.0002). There was no significant difference in length between cells expressing Nek2KD and Nek2 during disassembly (right panel, Fig. 1D). The KDs influenced cilia length but not cilia number suggesting that cilia length may be controlled by a different mechanism than initiation of cilia growth.

### One catalytically active kinase is sufficient to destabilize cilia when cilia are assembling but not disassembling

We next compared cilia number and length in cells expressing the kinase dead to those coexpressing the kinase dead with the wild-type companion kinase (compare AurAKD/Nek2 vs AurAKD; AurA/Nek2KD vs Nek2KD in Fig. 1) to determine whether the ability to depolymerize cilia requires both catalytically active kinases. If both active kinases are required for depolymerization, cilia would remain long and numerous in the double transfectants. Alternatively, the kinase dead enzymes could indirectly affect the activity of the wild-type partner enzyme by sequestering necessary factors. If the kinase dead protein appropriates negative factors that restrict kinase activity in cells assembling cilia, the companion kinase would be activated and cilia would disassemble. If, on the other hand, the KD sequesters positive factors that stimulate kinase activity in cells disassembling cilia, the kinase would be less able to depolymerize cilia and they would remain long and numerous. This model depends on AurA and Nek2 sharing a common set of upstream regulators.

Cells cotransfected with AurAKD/Nek2 or Nek2KD/AurA had significantly fewer and shorter cilia than the corresponding kinase dead when cilia were assembling (Fig. 1). Cells co-expressing AurAKD/Nek2 were significantly less ciliated than cells transfected with AurAKD alone and the control (AurAKD/Nek2 vs AurAKD, *p* = 0.02, Fig. 1A, left panel; AurAKD/Nek2 vs control, *p* < 0.0001). Cells co-expressing AurA/Nek2KD were significantly less ciliated than cells expressing Nek2KD alone and control (Fig. 1B, left panel, AurA/Nek2KD vs Nek2KD alone, *p* = 0.01; AurA/Nek2KD vs control, *p* < 0.0001) when cells are assembling cilia. Similarly, the cilia of cells co-expressing AurAKD/Nek2 were shorter than cells expressing AurAKD alone (Fig. 1C, left panel, *p* < 0.0001) and those co-expressing AurA/Nek2KD were shorter than cells expressing Nek2KD alone and control (Fig. 1D, left panel AurA/Nek2KD vs Nek2KD, *p* < 0.0001, AurA/Nek2KD vs control, *p* = 0.002).

Ciliation was not reduced in the double transfectants in cells disassembling cilia. Cilia number of the cotransfectants was significantly increased (compare AurAKD/Nek2 vs AurA KD; AurA/Nek2KD vs Nek2KD, Fig. 1A and B, right panels). The active enzymes could not reduce cilia number in the presence of the KD of the partner kinase (Fig. 1A and B, right panels). Cells coexpressing AurAKD/Nek2 were significantly more ciliated than those expressing AurAKD alone but not different from control (Fig. 1A, right panel, *p* = 0.02). AurAKD/Nek2 were significantly shorter than AurAKD alone but not different from control (Fig. 1C, right panel). There was no significant difference in cilia number or length between cells expressing AurA/Nek2KD and Nek2KD during disassembly. Together, these data demonstrate that the wild-type kinases can destabilize cilia in the presence of the catalytically inactive companion kinase when cilia are assembling but not disassembling. These data do not discriminate between a differential requirement for both kinases depending on cilia growth, or activation/inhibition of the companion kinase due to sequestration of common negative factors when cilia are assembling or positive factors when cilia are disassembling.

### The KD proteins enhance the activity of the partner wild-type kinase but only when cilia are assembling

To determine whether the KD might directly or indirectly affect the activity of the wild- type partner kinase, we compared cilia number and length between cells transfected with either wild-type kinase alone or the double transfectants (compare AurA/Nek2KD vs AurA; AurAKD/Nek2 vs Nek2, Fig. 1). Cells expressing AurA/Nek2KD were significantly less ciliated than cells expressing AurA alone or control when cilia are growing (Fig. 1A, left panel, AurA/Nek2KD vs AurA, *p* = 0.002; AurA/Nek2KD vs control, *p* < 0.0001). Similarly, AurAKD enhanced the depolymerization activity of Nek2 (Fig. 1B, left panel, AurAKD/Nek2 vs Nek2, *p* = 0.002). Only cells transfected with AurAKD/Nek2 displayed a decrease in cilia length compared to Nek2 during cilia assembly (Fig. 1D, left panel, *p* = 0.0001).

In contrast, coexpression with the kinase dead companion kinase did not result in increased depolymerization when cilia were disassembling compared to wild-type alone (Fig. 1A and B, right panels). Instead of stimulating depolymerization, the catalytically inactive kinase suppressed cilia depolymerization by the companion kinase. Cells dissembling cilia and coexpressing AurA/Nek2KD were significantly more ciliated than cells expressing AurA alone and control (Fig. 1A, right panel AurA/Nek2KD vs AurA, *p* = 0.002; AurA/Nek2KD vs control, *p* = .02). Cells coexpressing AurAKD/Nek2 were significantly more ciliated than Nek2 alone and control (Fig. 1B, right panel, AurAKD/Nek2 vs Nek2, *p* < 0.0004; AurKD/Nek2 vs control, *p* = 0.007). Cilia length was not significantly different in cotransfectants compared to wild-type or control.

Our results support differential effects of the KDs on the wild-type kinases depending on whether cilia are assembling or disassembling. Coexpression of the dominant negative enzyme with the wild-type companion kinase stimulates activity of the other kinase when cilia are growing but inhibits its activity when cilia are disassembling. We conclude from these experiments that the dominant negative enzymes sequester factors that inhibit depolymerization by the kinases when cilia are assembling and factors that stimulate depolymerization when cilia are disassembling. This result supports the existence of regulatory proteins common to the two kinases.

### AurA and Nek2 activities are additive when cilia are growing

**Fig. 2:**
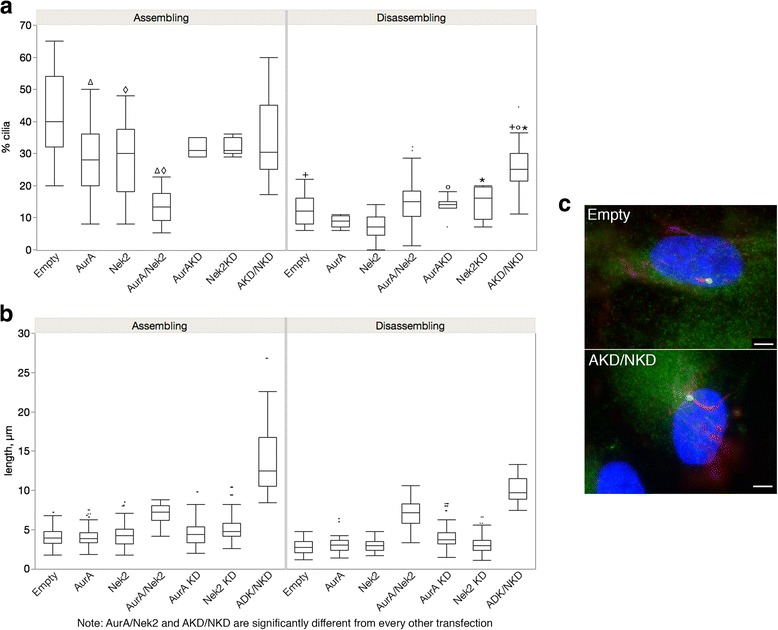
AurA and Nek2 activity is additive when cilia are growing. ARPE-19 cells were transfected with the following plasmids or combinations: AurA, Nek2, AurA/Nek2, AurAKD, Nek2KD, AurAKD/Nek2KD (AKD/NKD). Transfected cells were grown for 24 h then cilia assembly was induced by serum starvation (assembling, left panels) followed by reintroduction of serum to induce cilia disassembly (disassembling, right panels). Cilia number (**a**) and length (**b**) were measured for a total of 300 cells. Significant differences are indicated with similar symbols; *p* ≤ 0.05. AurA/Nek2 and AKD/NKD are significantly different from all other transfectants. (**c**) Cells transfected with either empty vector (top) or the AKD/NKD combination (bottom) were stained for gamma tubulin in green, acetylated tubulin in red, and DNA in blue. Scale bar equals 10 μm. The means for cilia number and length ± SEM are shown in Table 1

We next compared cells cotransfected with AurA/Nek2 or with AurAKD/Nek2KD (shown as AKD/NKD in the graph) to their respective single transfectants (Fig. 2). We predict that the double wild type transfectants would increase depolymerization compared to the single transfectants and that the double kinase dead would inhibit depolymerization more than the single KD transfectants. This was true for cells assembling cilia (Fig. 2A, left panel). AurA and Nek2 acted additively in cells assembling cilia to reduce the percentage of ciliated cells in cotransfected cells; cilia were significantly reduced in AurA/Nek2 double transfectants compared to single transfectants and control when cilia were assembling (Fig. 2A, left panel, AurA/Nek2 vs AurA or Nek2, *p* < 0.0001; AurA/Nek2 vs control, *p* < 0.0001). However, when cilia were disassembling, cilia were more numerous in double transfectants but this difference was not statistically significant nor different from control (Fig. 2A, right panel, *p* = 0.077 Nek2/AurA vs Nek2). These results suggest that their kinase activities are additive when cilia are assembling not disassembling suggesting that they have different downstream effectors that stimulate depolymerization independently of one another. The double KD increased ciliation in cells disassembling cilia compared to control (Fig. 2A right panel; AKD/AKD vs control, *p* < 0.0001) and compared to the KD of each kinase alone (AKD/NKD vs AKD, *p* < 0.0001; AKD/NKD vs NKD, *p* < 0.0001). No significant difference was observed during assembly. The effects of the double KD to stimulate ciliation suggests that AurA and Nek2, in addition to sharing activators as demonstrated by our wt/KD double transfections (Fig. 1), must have distinct coregulators as the KDs have additive effects to supresses depolymerization.

The effect of the double wild-type transfectants on cilia length was unexpected. Instead of shorter cilia as we would predict by the reduced cilia number in these samples (Fig. 2A), the double wild-type transfectants had significantly longer cilia compared to the single transfectants and controls (*p* < 0.0001) when cilia were either assembling (*p* < .0001) or disassembling (*p* < 0.0001) (Fig. 6B). One possibility is that the wild-type enzymes inhibit one another in control of cilia length. Alternatively, they could each regulate downstream negative effectors that control cilia length.

The effect on cilia length of the double KD was particularly striking (Fig. 2B). Cilia of double KD transfectants were extremely long compared to control and the single KD transfectants (*p* < 0.0001 in all cases). Cilia were about three times longer in cells transfected with AKD/NDK compared to controls when cilia were assembling and disassembling. The surprising finding that the double KD causes extremely long cilia (Fig. 2C) suggests to us that repression of multiple pathways is necessary to maintain cilia length. This is consistent with the additive effects of the KD enzymes.

### Nek2 and AurA form a complex in ARPE-19 cells

**Fig. 3:**
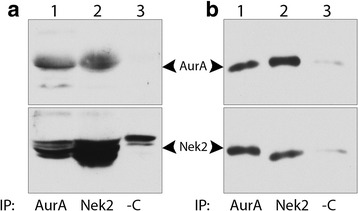
Nek2 and AurA form a complex in ARPE-19 cells. (**a**) Cells were cotransfected with plasmids expressing AurA and Nek2 and cell lysates immunoprecipitated with either AurA antibodies (lane 1), Nek2 antibodies (lane 2), or no antibody (land 3) and probed for either AurA or Nek2. (**b**) Untransfected ARPE-19 cell lysates were immunoprecipitated with either AurA (lane 1), Nek2 (lane 2), or no antibody and probed for either AurA or Nek2. Each immunoprecipitate was blotted for the immunoprecipitating antibody to verify immunoprecipitation

To determine whether Nek2 and AurA can interact with one another in cultured cells we coexpressed AurA and Nek2 in ARPE-19 cells and immunoprecipitated the complex with antibodies to either Nek2 or AurA followed by interrogation of the complex with antibodies specific for the other kinase. Complexes immunoprecipitated with AurA contain Nek2 and reciprocally (Fig. 3A). The endogenous kinases are also found associated with one another (Fig. 3B). This suggests that the kinases may directly interact to regulate one another during cilia assembly and disassembly. This is consistent with the results described in Fig. 1 where we show that the kinases functionally interact to disassemble cilia. Alternatively, an intermediary protein could be responsible for the effects we observe.

### AurA inhibition increases cilia number

**Fig. 4:**
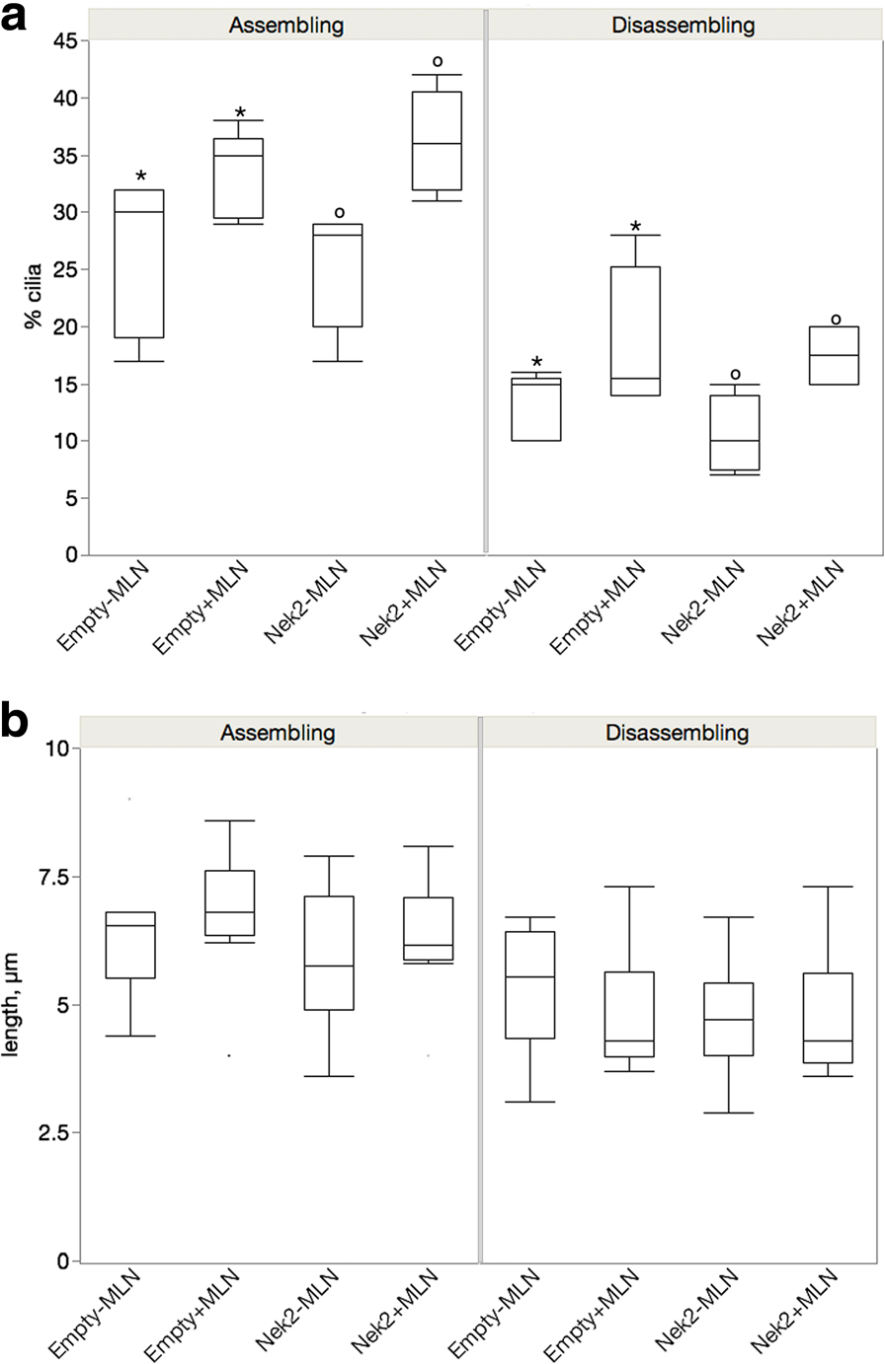
AurA inhibition increases cilia number. ARPE-19 were tranfected with control vector or the Nek2 expression plasmid in the presence or absence of 0.20 μM AurA inhibitor MLN8237, cells were serum starved to stimulate cilia formation (assembling, left panels) followed by reintroduction of serum to induce cilia disassembly (disassembling, right panels). Cilia number (**a**) and length (**b**) were measured for a total of at least 300 cells. Significant differences are indicted with similar symbols, *p* ≤ 0.05. The means for cilia number and length ± SEM are shown in Table 1

To determine whether the wild type kinases effect the activity of the other and whether the kinases have distinct roles in ciliation, we compared cilia number and length in cells transfected with the active kinase while inhibiting the partner kinase with a small molecule inhibitor (Fig. 4). If AurA affects Nek2 when cilia are assembling, release of this action by inhibition of AurA’s kinase activity will alter Nek2 catalyzed cilia depolymerization resulting in fewer and shorter cilia. Instead, incubation of cells assembling cilia and expressing Nek2 with the small molecule inhibitor of Aurora A, MLN8237 (MLN) [30], resulted in an increase in the percentage of ciliated cells (Fig. 4A, left panel, Nek2 + MLN vs Nek2 − MLN *p* = 0.001). We also observed this effect in cells disassembling their cilia (Fig. 4A, right panel, Nek2 + MLN vs Nek2 − MLN, *p* = 0.04). However, this appears to be an effect of MLN alone since this difference was also seen when comparing treated control transfected cells with untreated cells (Fig. 4A, empty − MLN vs empty + MLN, *p* = 0.01). Treatment of Nek2 transfected cells with MLN did not effect cilia length when cilia were either assembling or disassembling (Fig. 4B). Therefore, any effect of inhibition of AurA on Nek2 activity is masked by the general effect of the AurA inhibitor on cilia number. Our results demonstrate that AurA has a more significant role in control of cilia nucleation than length.

### Inhibition of Nek2 significantly lengthens cilia when cilia are assembling and disassembling

**Fig. 5:**
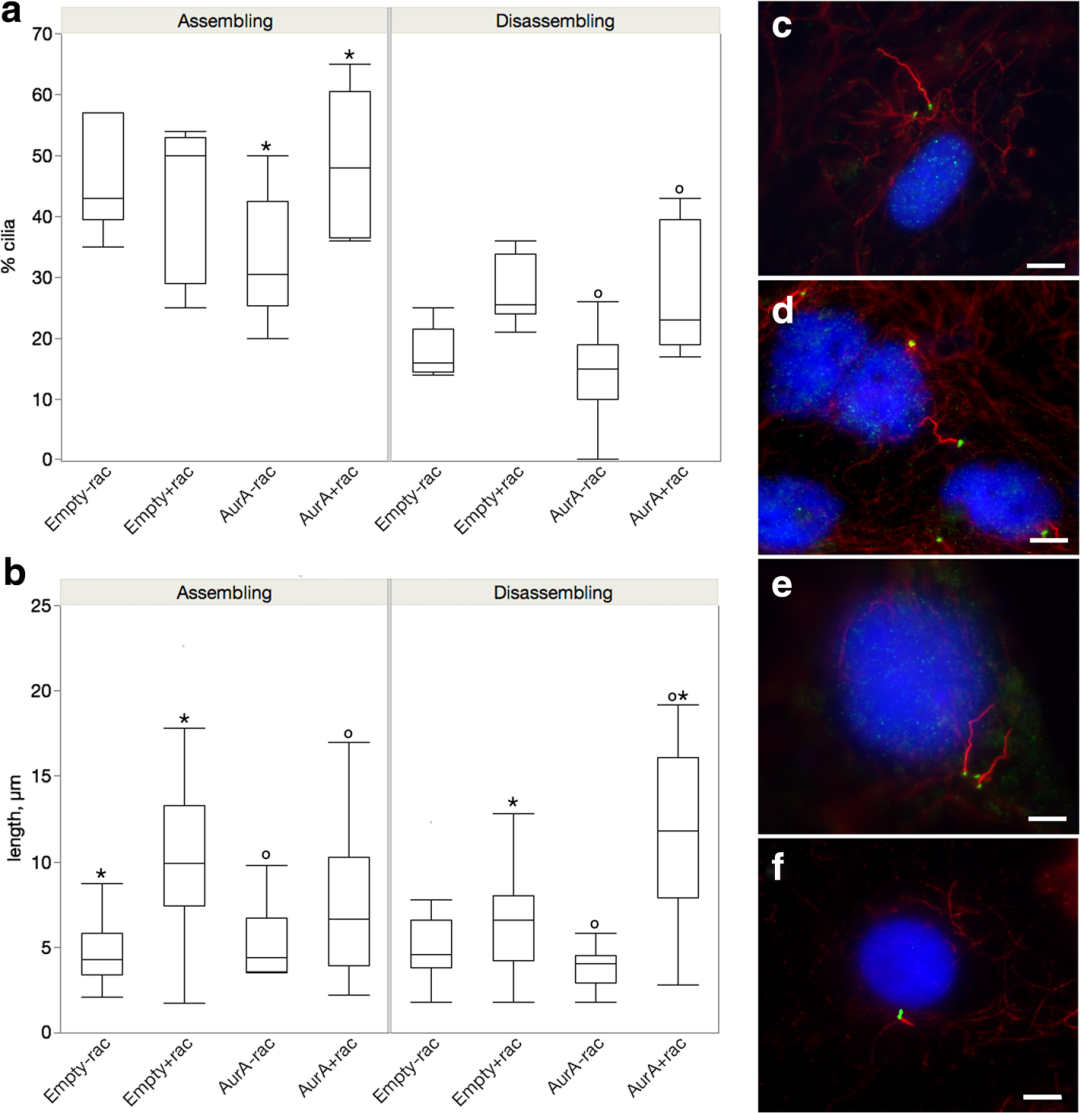
Inhibition of Nek2 significantly lengthens cilia when cilia are assembling and disassembling. 10 μM of the Nek2 inhibitor (rac-CCT250863) was added to the AurA transfections, cells were serum starved to stimulate cilia formation (assembling, left panels) followed by reintroduction of serum to induce cilia disassembly (disassembling, right panels). Cilia number (**a**) and length (**b**) were measured for a total of at least 300 cells. (**c-e**) show typical examples of long cilia in cells serum starved and exposed to the Nek2 inhibitor and stained for gamma tubulin (green), acetylated tubulin (red) and DNA (blue); (**f**) shows a control cell. Scale bar equals 10 μm. The means for cilia number and length ± SEM are shown in Table 1

We next treated cells transfected with either empty vector or AurA with the Nek2 inhibitor rac-CCT 250863 (termed here as rac, Fig. 5) [31]. Treatment of control transfected cells had no effect on ciliation when cilia were assembling or disassembling. However, cells overexpressing AurA and treated with rac were significantly more ciliated than untreated AurA overexpressing cells when cells were assembling or disassembling (Fig. 5A, *p*=0.005 in both cases). Therefore, inhibition of Nek2 counteracts the effect of AurA indicating that Nek2 may be upstream of AurA or in a parallel pathway in control of cilia number. The effect of the Nek2 inhibitor on cilia length was striking. Cilia were significantly longer in AurA transfected cells inhibited for Nek2 when cells were both assembling or disassembling (Fig. 5B, AurA − rac vs AurA + rac, assembling, *p *= 0.001, disassembling, *p* < 0.0001). Inhibition of Nek2 increased the length of cilia in control transfected cells when cilia were assembling their cilia (*p* ≤ 0.0001). Examples of long cilia are shown in panels C-E (Fig. 5). In many cells with multiple centrosomes, likely a consequence of Nek2 inhibition, we observed more than one cilia in an individual cell (Fig. 5E).

The effect on cilia length in cells assembling cilia appeared solely due to Nek2 inhibition as there was no difference between control cells treated with inhibitor and AurA transfectants treated with inhibitor; however, in cells disassembling cilia, cilia were significantly longer in cells inhibited for Nek2 and transfected with AurA than treated cells transfected with empty vector or untreated cells expressing AurA (Fig. 5B, right panel, *p* = 0.0001 for both). As seen for cilia number, Nek2 inhibition counteracts the effect of AurA to destabilize cilia in cells disassembling cilia. We conclude that Nek2 has a profound effect on cilia length and its action is independent of AurA expression in cells assembling cilia but AurA overexpression further inhibits cilia depolymerization when cells are already disassembling their cilia. In this case, Nek2 may activate AurA because in its absence AurA is less able to depolymerize cilia when cilia are disassembling.

### R42 overexpression increases the number and length of cilia

**Fig. 6:**
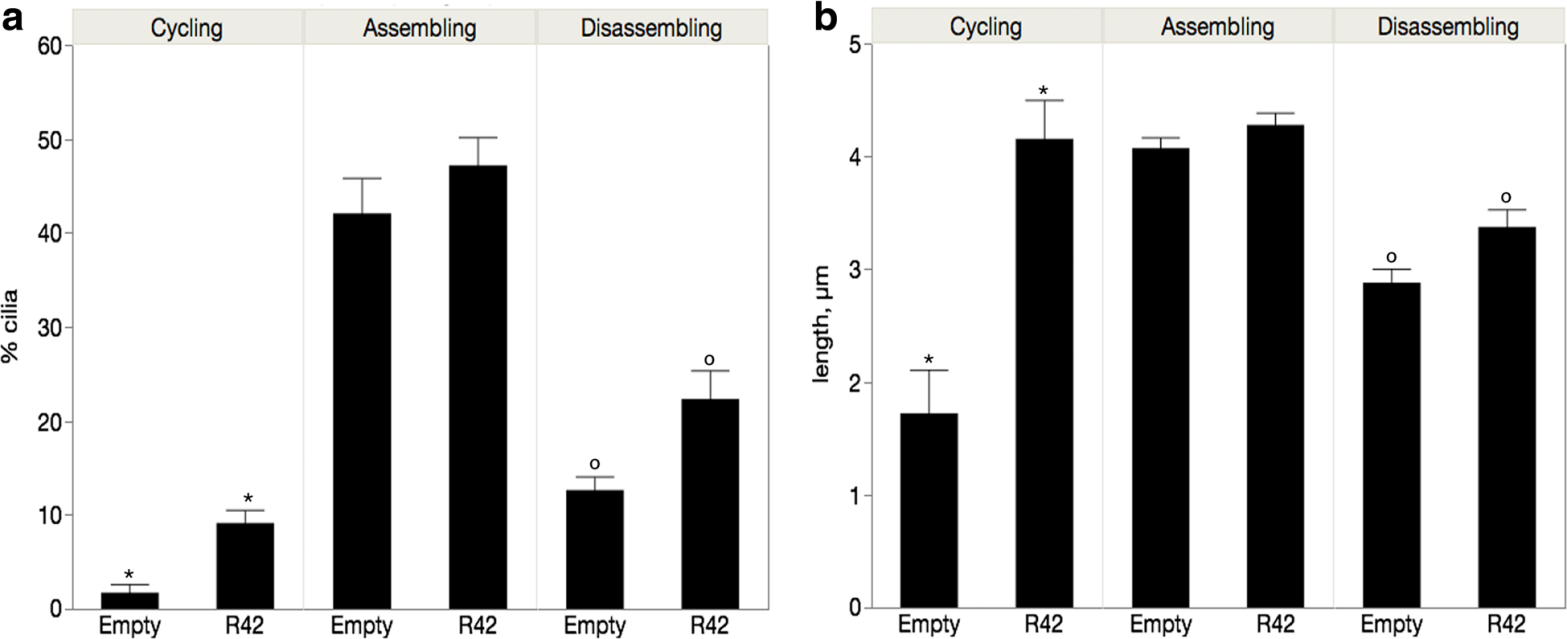
PPP1R42 overexpression induces an increase in cilia number and length. ARPE-19 cells were transfected with expression vector containing FLAG-tagged PPP1R42 (R42), grown for 24 h (cycling, left panel), induced to assemble cilia by removal of serum for 48 h (assembling, middle panel) followed by reintroduction of serum to cause cilia to disassemble (disassembling, right panel). Cilia number (**a**) and length (**b**) were measured at each condition. Transfection with empty vector was the negative control. A total of 300 cells were counted and significant differences are indicated with matching symbols; *p* ≤ 0.05. The means for cilia number and length ± SEM are shown in Table 1

We have previously identified PPP1R42 as a binding partner for PP1 in the testes that participates in centrosome dynamics [24]. PPP1R42 (R42) is associated with activated PP1 and localized to the base of flagella in spermatids and cilia in ARPE-19 cells [24, 32]. PP1 is known to regulate both AurA and Nek2 in centrosome separation [19–22], therefore we sought to determine whether R42 could regulate ciliation in ARPE-19 cells and whether any effect was dependent on cilia growth. R42 was overexpressed in actively dividing cells (cycling), and cells where cilia were assembling or disassembling (Fig. 6). R42 overexpression was confirmed by western blot (Additional file 1: Figure S1D) and indirect immunofluorescence (Additional file 2: Figure S2). Transfection with empty FLAG vector served as negative control. Cells overexpressing R42 were significantly more ciliated compared to the negative control when cells were actively cycling or when cilia were disassembling but not when cilia were assembling (Fig. 6A). There was a 5-fold increase in the percentage of ciliated cells in the cycling cell population overexpressing R42 compared to control (Fig. 6A, left panel, *p* = 0.04); and an 83% increase when cilia were disassembling (Fig. 6A, right panel, *p* = 0.03).

Cilia length was also responsive to R42 overexpression when cells were cycling or disassembling cilia, but not when cilia were assembling. Cilia were 100% longer than control cells (Fig. 6B, left panel, *p* < 0.0001) when R42 was overexpressed in a population of dividing cells and 17% longer than control (Fig. 6B, right panel, *p* = 0.03) when cilia were disassembling. Like our results with AurA and Nek2, we see different effects of overexpression on cilia depending on growth status. R42 overexpression does not increase cilia number and length when cilia are assembling, suggesting that cilia number and length are at a maximum in these cells, as we concluded for the KD coexpressions (Fig. 1). However, our results with the double KD (Fig. 2) demonstrated that cilia can become very long when both AurA and Nek2 are affected.

### Depletion of PPP1R42 reduces cilia number and length and produces deformed cilia

**Fig. 7:**
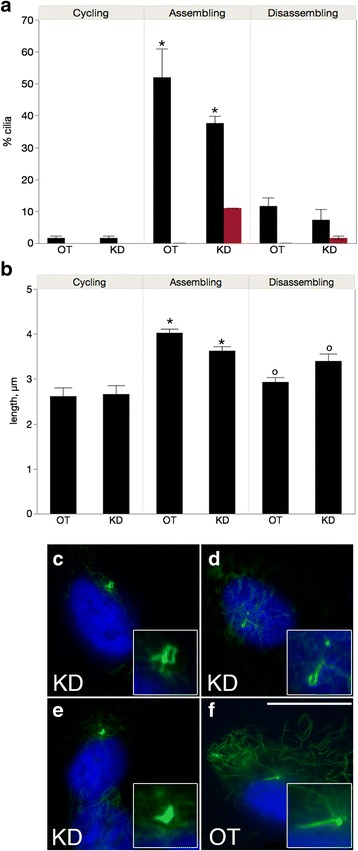
PPP1R42 depletion reduces cilia number and length and produces abnormal cilia. (**a**) ARPE-19 cells were treated with either PPP1R42 targeting siRNA (knockdown, KD) or with off-target siRNA (off target, OT) and grown for 24 h (cycling, left panel), then cilia assembly was induced by serum starvation (assembling, middle panel) following reintroduction of serum to induce cilia disassembly (disassembling, right panel). Cilia number and length (**a** and **b**) were measured in each condition, 300 cells were counted. Significant differences are indicated with similar symbols; *p* ≤ 0.05. Percentage distorted cilia is shown by the red bars in panel A. (**c**-**e**) ARPE-19 cells were treated with R42 KD siRNA for 24 h and three representative cells are shown with cilia stained green with anti-acetylated tubulin antibody (enriched in cilia) and nuclei blue with DAPI. Insets show deformed cilia at higher magnification. (**f**) A representative control cell treated with off-target siRNA. Scale bar = 10 μm. The means for cilia number and length ± SEM are shown in Table 1

Protein levels of R42 were reduced with an siRNA pool to examine the effect of this PP1 binding protein on cilia (Additional file 1: Figure S1C and Fig. 7). We have used this approach successfully to show that R42 functions in centrosome dynamics [24]. Cilia length and number were measured in treated cells that were actively dividing, assembling or disassembling cilia. R42 knockdown caused a decrease in ciliated cells but this difference was significant only when cilia were growing (Fig. 7A, p=0.001, middle panel). R42 knockdown significantly decreased cilia length compared to control when cilia were growing; however, cilia were longer in R42 depleted cells that were disassembling cilia (*p* = 0.001 and *p* = 0.006, respectively, Fig. 7B). We note that overexpression of R42 also increased length of cilia instead of decreasing it. This may be because R42 is a multifunctional protein binding to PP1 and Nek2 and these enzymes may have complex interactions in control of cilia length. Eleven percent of all cells assembling cilia displayed deformed cilia while a smaller fraction of cilia (1.7%) were deformed when cilia were disassembling (Fig. 7A, red bars). Deformed cilia were never seen in control cells (Fig. 7F). The malformed cilia demonstrated a range of shapes from curled into a sphere (Fig. 7B and D) to hook-shaped (Fig. 7C). This difference between cells assembling or disassembling their cilia is similar to our kinase/kinase dead coexpression studies above that revealed different effects on cilia number and length depending on cilia growth status.

### R42 interacts with Nek2 and inhibits its activity in vitro

**Fig. 8:**
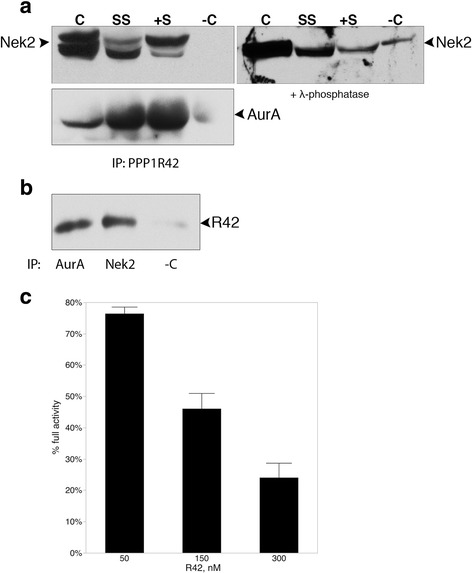
PPP1R42 interacts with Nek2 and inhibits Nek2 activity in vitro. (**a**) PPP1R42 was overexpressed with either Nek2 or AurA in ARPE-19 cells, grown for 24 h (C, cycling) then the cells were induced to assemble cilia (SS, serum starved) followed by disassembly (+S, serum added back). Protein complexes were immunoprecipitated with anti-PPP1R42 antibody, or no antibody as negative control (−C), and the blots probed for Nek2 or AurA. Immunoprecipitates were treated with λ-phosphatase and probed with Nek2 antibody (**a**, right panel). (**b**) Protein complexes were immunoprecipitated from untransfected ARPE-19 cells with anti-AurA and anti-Nek2 antibodies and probed for PPP1R42. (**c**) Nek2 kinase activity was assayed in the presence of 50, 150 and 300 nM recombinant R42 protein and activity calculated as a percentage of untreated Nek2 activity

To determine whether R42 interacts with these kinases during cilia assembly and disassembly, we coexpressed R42 with each kinase in cycling cells or in cells with assembling or disassembling cilia, then immunoprecipitated with an R42 antibody and probed for the presence of Nek2 (Fig. 8A, top) or AurA (Fig. 8A, bottom). R42 associates with both Nek2 and AurA during all growth conditions. Notably, R42 interacts with different isoforms of Nek2 depending on the growth status of cilia. Both isoforms interact equally with R42 in dividing cells while the complex with the faster migrating Nek2 species is more abundant when cilia are assembling (Fig. 8A, SS left panel) and the complex with the slower migrating Nek2 species is more abundant when cilia are disassembling (Fig. 8A, +S left panel). Alternative splicing of the *nek2* gene results in isoforms Nek2A, Nek2B, and Nek2C (42 to 48kD) [33]. We used the Nek2A expression plasmid provided by the Fry lab for our experiments; therefore, only one splice variant is overexpressed in cells. Treatment of the R42 IP with λ-phosphatase collapses all bands to the faster migrating species supporting phosphorylation as responsible for the observed isoforms (Fig. 8A, right). Immunoprecipitation of endogenously expressed proteins isolated from untransfected cells showed that R42 interacts with both kinases in vivo (Fig. 8B). To determine whether R42 has any effect on Nek2 activity, we conducted kinase assays after incubating Nek2 with recombinant R42 at different concentrations. We observed a dose dependent inhibition of Nek2 activity by R42 indicating direct interaction and inhibition of Nek2 by R42 (Fig. 8C). R42 also interacts with PP1 [32]; therefore, we wanted to determine whether R42 might also directly affect PP1 activity. We conducted phosphatase assays using recombinant PP1 and R42, along with the PP1 inhibitor PPP1R2 (Inhibitor-2; [34]). Recombinant R42 did not affect PP1 activity whereas PPP1R2 was able to repress phosphatase activity, as has already been shown by others (Additional file 3: Figure S3) [35]. R42 may target PP1 to the centrosome for interaction with kinases.

### Nek2 and AurA counteract the effects of R42 overexpression on cilia

**Fig. 9:**
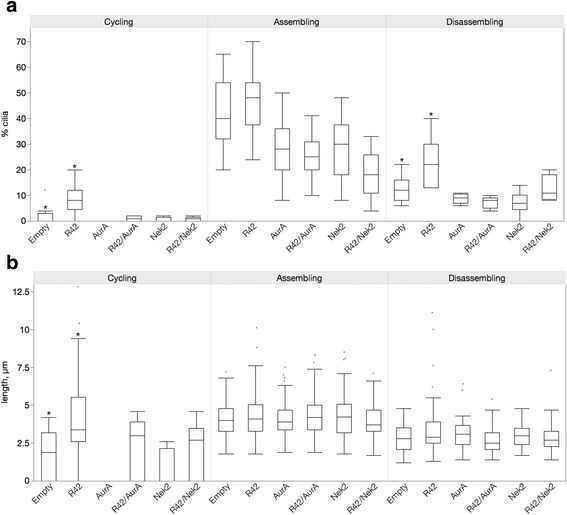
AurA and Nek2 counteract the stimulatory effect of PPP1R42 on cilia. ARPE-19 cells coexpressing PPP1R42 (R42) with either kinase (R42/Nek2; R42/AurA), or expressing either kinase alone were grown for 24 h (cycling, left panel), then cilia assembly was induced by serum starvation (assembling, middle panel) following by reintroduction of serum to induce cilia to disassemble (disassembling, right panel). Transfection with empty vector served as negative control. Cilia number (**a**) and length (**b**) were measured at each condition; 300 cells were counted. Significant differences are indicated with asterisks; *p* ≤ 0.05. The means for cilia number and length ± SEM are shown in Table 1

We have shown that R42 inhibits Nek2 directly and is a PP1 binding protein [32]. Both Nek2 and AurA kinases induce cilia disassembly and are negatively regulated by PP1 [8, 13, 19, 20, 24, 29, 36, 37]. To determine whether R42 could reverse the depolymerizing activity of Nek2 or AurA, we coexpressed either kinase with R42 (Fig. 9, Additional file 1: Figure S1A). Under every growth condition, R42 was not able to significantly increase the low percentage of ciliated cells induced by either kinase alone (Fig. 9A). A similar effect on cilia length was seen; R42 coexpression with either kinase did not lengthen cilia (Fig. 9B).

## Discussion

**Fig. 10:**
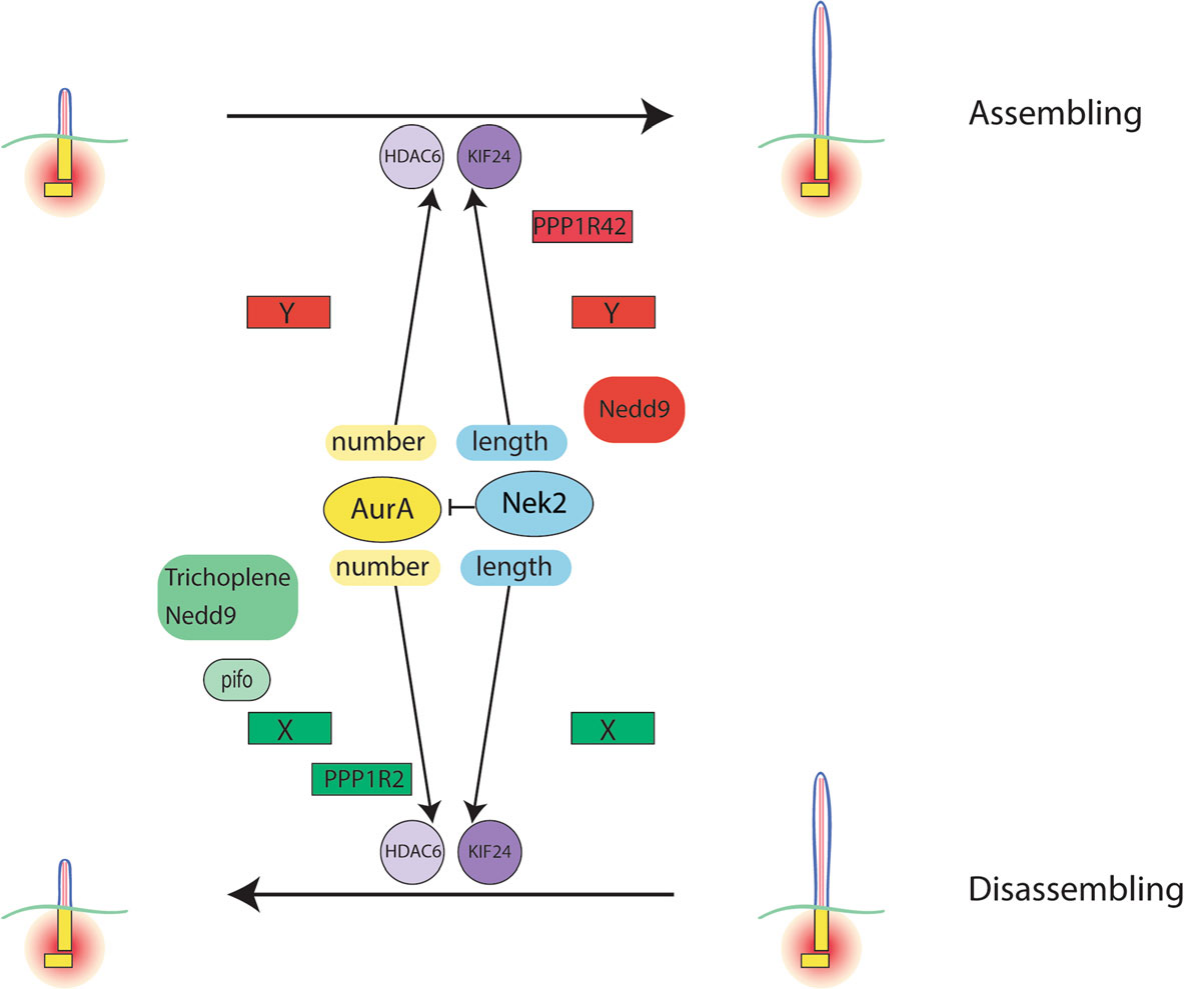
Positive and negative factors differentially regulate AurA and Nek2. Positive regulators that stimulate depolymerization by AurA and Nek2 when cilia are disassembling are shown in green while factors that counteract depolymerization are shown in red. The downstream targets of the kinases, HDAC6 and KIF24 are shown in shades of purple. The letters X and Y indicated common but unknown negative and positive regulators. Negative regulation of AurA by Nek2 is indicated

The mechanisms governing cilia shortening are not well understood. AurA and Nek2 destabilize cilia prior to entry of cells into G_0_ [8, 10, 11, 13, 38–41]. The molecules govering this disassembly are depicted in the cartoon in Fig. 10. AurA is activated by multiple intersecting pathways including NEDD9 (Hef1), a scaffolding protein associated with focal adhesions [13] and trichoplein which activates AurA to prevent aberrant cilia formation in proliferating cells [38]. An additional upstream activator, pitchfork, activates AurA at the embryonic node [39]. Nedd9 also regulates Nek2 but inhibits its activity rather than activating this enzyme [42]. AurA and Nek2 also have unique and overlapping downstream effectors. Both kinases can activate HDAC6 to depolymerize microtubules; however, Nek2 can also activate the kinesin KIF24, a member of the family of depolymerizing kinesins, to stimulate cilia depolymerization independent of AurA [11, 13]. Inhibition or overexpression of Nek2 does not affect phosphorylation and activation of AurA at the centrosome, suggesting that Nek2 is downstream of AurA or in a parallel pathway to stimulate cilia absorption [8, 10]. This result examined kinase activation prior to mitosis and not cilia behavior. Our results that Nek2 inhibition can reverse the destabilization effect of AurA to regulate cilia number and length (Fig. 5) indicate that Nek2 may act upstream or in a parallel pathway to control cilia.

In these studies, we provide evidence that AurA and Nek2 functionally and physically interact in cilia reabsorption in ARPE-19 cells and that this interaction varies with the growth state of cilia. The wild-type enzyme can destabilize cilia in the presence of the dominant negative partner enzyme but only when cilia are assembling not disassembling (Fig. 1). One interpretation of this result is that one enzyme is dispensable when cilia are growing but not shortening. This may reflect the fact that the kinases are not as active when cilia are assembling and therefore overexpression of only one kinase is sufficient to shift the balance from increased to reduced ciliation. When cilia are shortening, both enzymes are required to further shorten cilia suggesting that the rapid depolymerization necessary for reentry into the cell cycle may require maximal activation of multiple pathways through activation of several downstream effectors by both Nek2 and AurA.

Alternatively, the dominant negative could have an indirect effect on the activity of the partner kinase. We predict that the kinases are restrained by negative factors when cilia are growing to prevent premature destabilization of cilia in G_0_. The dominant negative proteins sequester these negative factors from the partner kinase thereby activating it to prematurely destabilize cilia. Similarly, we predict that the kinases are activated in cells disassembling cilia to efficiently depolymerize cilia prior to reentry into the cell cycle, therefore, competition for positive factors by the dominant negative partner kinase inhibits the other kinase and blocks depolymerization.

This latter explanation is supported by our experiments comparing cells expressing the wild-type kinases to those coexpressing the kinase dead with the wild-type partner kinase (Fig. 1). The dominant negative of either kinase potentiated depolymerization compared to the wild-type kinase alone when cilia were growing and countered depolymerization when cilia were shortening. This is consistent with competition of the dominant negative protein for negative factors when cilia are assembling and positive factors when cilia are disassembling. One conclusion from this finding is that AurA and Nek2 must share common activators and inhibitors of cilia disassembly. However, only reciprocal regulators have been reported. AurA at the basal body is positively regulated by the focal adhesion scaffolding protein Nedd9 to induce cilia disassembly when cells reenter the cell cycle [13]. Conversely, Nedd9 inhibits Nek2 activity to disassemble cilia and increase pericentriolar material at the centrosome [42]. The opposing activity of Nedd9 on Nek2 and AurA is counterintuitive to their demonstrated roles in disassembling cilia and would not likely counteract one another [8, 10, 11, 13]. It is important to note that the report concerning Nek2 inhibition by Nedd9 was not obtained from cells disassembling cilia but from cycling cells and may reflect differential regulation in cycling vs disassembling cells which is consistent with our work [42]. No common activators or inhibitors of AurA and Nek2 have been identified, however, we would predict that associated positive regulatory factors would be enriched in cells disassembling cilia and negative regulators in cells assembling cilia. This functional interaction is consistent with our finding that the kinases are associated with one another (Fig. 3) and the work of others demonstrating that the two kinases colocalize to the basal body [8, 42].

Although Nek2 and AurA appear to share regulatory proteins in common, their inhibition has different effects on cilia number and length. We show that AurA plays an important role in regulating cilia number (Fig. 4) while Nek2 inhibition significantly impacts cilia length (Fig. 5). This suggests that nucleation of cilia is inhibited by AurA in cells either assembling or disassembling cilia while Nek2 functions primarily to maintain the steady state length of cilia. Our finding contrasts with a previous result that flagella length in *Clamydomonas reinhardtii* is regulated by an AurA-like protein [16]. However, our experiments examine cilia length under dynamic rather than steady state conditions that may involve different mechanisms of regulation. Additionally, we show that Nek2 activates AurA in control of cilia number because Nek2 inhibition inhibits depolymerization by AurA (Fig. 5). However, our results with the double wild-type transfectants indicate that the functions of the kinases are overlapping. Cells cotransfected with active AurA and Nek2 had significantly fewer cilia compared to each kinase transfected separately when cilia were assembling; however, cilia length was increased in the double wild-type transfection. When cilia are disassembling and the kinases are activated, expression of both dominant negative kinases suppresses depolymerization and increases both cilia number and length above the single KD alone but only when cilia are disassembling. This suggests that the mechanism of AurA and Nek2 in regulation of cilia length differs from their control over cilia number.

A particularly intriguing finding was our observation that cilia were extremely long in cells transfected with AKD/NKD (Fig. 2). HDAC6 is a downstream effector of both Nek2 and AurA, therefore we would expect that axonemal microtubules might be stabilized in the double KD and that this effect would be augmented compared to the single KD transfectants [10, 13]. Furthermore, the depolymerizing kinesin KIF24 is activated by Nek2 to ensure cilia disassembly and the Nek2-KIF24 pathway is temporally and functionally distinct from the AurA-HDAC6 pathway [11]. We would expect interruption of both pathways to be additive. Ciliary growth is mediated by intraflagellar transport (IFT) via anterograde motors that move protein and membrane to the ciliary tip and retrograde motors that return cargo to the cilia base [43]. Cilia length is in a state of “dynamic stability” with assembly in balance with disassembly [44–46]. One explanation of our result is that by increasing the length of the microtubule “track” upon which motors transport cargos to the ciliary tip, the cilia is extended beyond its steady state length. This indicates that the length of the microtubule track is one of the limiting factors for IFT. Cilia length in *Chlamydomonas reinhardtii* is regulated by phosphorylation of CALK, an AurA-like protein. Inhibition of CALK activity increases flagella length to twice their normal length [47]. In our system, Nek2 has a more profound effect on cilia length than does AurA.

Nek2 is proposed to act as a switch between cilia growth and resorption in the establishment of left right asymmetry [10]. Increased amounts of Nek2 shift the balance to cilia depolymerization while decreased Nek2 causes centrosome defects; therefore, it is proposed that Nek2 promotes cilia biogenesis and homeostasis. We have identified a new inhibitor of Nek2, the PP1 binding protein PPP1R42. R42 inhibits the activity of Nek2 in vitro and binds to Nek2 in cultured cells. This is consistent with our finding that R42 overexpression increases ciliation presumably by inhibiting cilia depolymerization by Nek2. We conclude that R42 is partially responsible for inhibition of Nek2 in addition to dephosphorylation by PP1 [20, 22]. Our data establishes that different phosphorylated isoforms of Nek2 interact with PPP1R42 depending on cilia growth state and further supports our proposal that Nek2 has different activities when cilia are assembling or disassembling.

We have previously shown that R42 interacts with PP1; however, R42 does not directly affect PP1 activity in vitro (Additional file 3: Figure S3). R42 may target PP1 to Nek2 to facilitate dephosphorylation and inhibition of Nek2; experiments are planned to determine whether a ternary complex exits containing Nek2, PP1, and R42.

## Conclusions

Figure 10 presents a model for cilia assembly (top) and disassembly (bottom) based on this work and that of others. In this diagram, positive factors that stimulate depolymerization are shown in green and negative regulators in red and flank either AurA or Nek2. Factors identified by others are Nedd9, trichoplein, pitchfork (pifo) and PP1R2 [10, 13, 38, 48]. HDAC6 and KIF24 are downstream targets of AurA and Nek2 that act to destabilize the microtubules of the axoneme [11, 13]. The work described here makes several unique contributions. First, we propose that AurA and Nek2 are activated and inhibited by positive (green) and negative (red) regulators which control AurA and Nek2 activity differently depending on the growth state of cilia. We predict that depolymerization is restrained by negative factors that are active when cilia are assembling (top) and that polymerization is enhanced by positive factors when cilia are disassembling (bottom). Second, AurA and Nek2 exist in a complex and share positive and negative regulators, indicated by unknown factors X and Y. This conclusion is supported by our data that the kinase dead of one enzyme affects the activity of the other presumably by sequestering regulatory factors (Fig. 1). Third, AurA and Nek2 have distinct effects on cilia number and length; AurA inhibition increases cilia number with no effect on cilia length while Nek2 inhibition increases cilia length (Figs. 4 and 5).

Fourth, the depolymerizing ability of AurA and Nek2 is additive when cilia are assembling. In addition, the double wild-type transfectants had longer but fewer cilia (Fig. 2). Finally, we show that PPP1R42, a PP1 binding protein, binds to and inhibits Nek2 in vivo and increases ciliation when cilia are shortening but not growing. This dependence on cilia growth status mirrors our results with the kinases. PPP1R42 may actively promote assembly when cilia are disassembling or prevent cilia from shortening. We also show that PPP1R42 does not activate PP1 suggesting that its inhibition of Nek2 is independent of its interaction with PP1.

This work establishes a complex web of regulation of cilia depolymerization that involves both positive and negative effectors that are differentially regulated in cells assembling cilia compared to disassembling cilia. We have also identified a new negative regulator of Nek2 which does not act through PP1 but binds directly to the kinase and represents a candidate molecule for differential modulation in cells assembling or disassembling cilia.

## Methods

### Cell culture and nucleic acid transfection

Human retinal pigmented epithelial cells (ARPE-19) were obtained from American Type Tissue Collection (Manassas, VA) and grown in DMEM-F12 media supplemented with 10% fetal bovine serum and 1% penicillin-streptomycin as directed by the supplier. To drive cells into quiescence, cells were washed three times in PBS and grown in culture media without fetal bovine serum for 48 h. To release cells from the quiescence, 10% serum was added back to the culture media for 24 h. To study the effect of protein overexpression on cilia growth and retraction, cells were transfected with expression vectors encoding PPP1R42, AurA, AurA kinase dead mutant, Nek2, or Nek2 kinase dead mutant, and 24 h later serum was removed from the media to halt growth and drive cells into G_0_ to stimulate cilia growth. Following incubation in serum free media for 48 h, serum was added to the growth media to induce cilia disassembly and cells were observed for cilia number and length 24 h later. Protein expression was confirmed by western analysis (Additional file 1: Figure S1).

Overexpression of PPP1R42 in ARPE-19 cells was performed using a PPP1R42-FLAG expression vector constructed by insertion of the PPP1R42 cDNA downstream and in frame with the FLAG tag of 3XFLAG CMV-14 (Sigma-Aldrich; St. Louis, MO). Overexpression of Nek2 was performed using a Nek2-myc expression vector while inhibition of Nek2 activity was achieved using a Nek2-K37R-myc expression vector, a dominant negative form of Nek2 (kind gifts of Dr. Andrew Fry, [20]). Overexpression of AurA was performed using an AurA-myc expression vector while inhibition of AurA kinase activity was achieved using an AurA-K162R-myc expression vector, a dominant negative form of AurA (kind gifts of Dr. Erich Nigg, [26]). Cells were transfected using Lipofectamine 2000® (Life Technologies; Lincoln, NE) according to the manufacturer’s recommendations. Briefly, cells were transfected for 24 h and overexpression was confirmed by western blot (Additional file 1: Figure S1). 90–95% transfection efficiency of the FLAG plasmids was determined using immunofluorescence. Depletion of PPP1R42 in ARPE-19 cells was performed using the ON-TARGETplus SMARTpool siRNA LOC286187 from Dharmacon/Thermo Scientific (Pittsburgh, PA) as previously described [24]. Cells were transfected using the Lipofectamine-RNAiMAX reagent (Life Technologies; Lincoln, NE) according to manufacturer’s recommendations. Briefly, cells were transfected for 48 h with a final concentration of 20 μM siRNA. Following incubation, knockdown was confirmed by western blot (Additional file 1: Figure S1).

The AurA inhibitor MLN8237 (0.25 μm; Selleckchem, Houston, TX) was added to cells transfected with the Nek2 plasmid, grown for 24 h, serum removed to induce cilia formation for 48 h and serum reintroduced to induce cilia disassembly. This procedure was repeated with the AurA plasmid and 10 μm Nek2 inhibitor (rac-CCT 250863, Tocris Biosciences, Bristol, UK).

### Western blot

ARPE-19 total cell lysates were prepared as previously described [24]. Briefly, cells were resuspended in cell lysis buffer (50 mM Tris, pH 7.5, 1 mM EDTA, 1 mM EGTA, and 1% NP-40) with protease and phosphatase inhibitors. Protein was separated using SDS-PAGE and proteins were transferred from the gel to polyvinylidene difluoride (PVDF) membrane (BIO-RAD Laboratories; Hercules, CA). PPP1R42-FLAG was detected using anti-FLAG antibody (1:1000; F1804; Sigma-Aldrich; St. Louis, MO) and confirmed with anti-human PPP1R42 antibody (1:1000; HPA028628; Sigma-Aldrich; St. Louis, MO). Nek2-myc and AurA-myc were detected using anti-myc antibody (1:1000; TA325701; Origene Technologies; Rockville, MD). Immune complexes bound to the membrane were detected with horseradish peroxidase-conjugated donkey secondary antibody (711–035-152; Jackson ImmunoResearch Inc.; West Grove, PA) and developed with SuperSignal® West Pico Chemiluminescent Substrate according to directions of the manufacturer (Thermo Fisher Scientific; Asheville, NC).

### Indirect immunofluorescence

ARPE-19 cells were grown on coverslips, fixed and permeabilized with methanol at −20 °C for 10 min, and then non-specific sites were blocked by incubation with 3% BSA in Tris-buffered saline and Triton X-100 (TBST) (20 mM Tris, pH 7.5, 150 mM NaCl, 2 mM EGTA, 0.1% Triton X-100) for 30 min. The cells were incubated with anti-acetylated-tubulin antibody to detect primary cilia (1:1000; T6793; Sigma-Aldrich; St. Louis, MO). Acetylated tubulin is a well-accepted marker for primary cilia. Cells were then incubated with FITC-conjugated donkey anti-mouse secondary antibody (1:200; 715–095-150; Jackson ImmunoResearch Inc.; West Grove, PA). DNA was stained with 4′,6-diamidino-2-phenylindole (DAPI) incorporated into the mounting media (Vector Labs; Burlingame, CA). The intracellular localization of proteins was observed with a Nikon E600 fluorescence microscope, Pan Fluor 100× objective (N.A. 0.5–1.3) or Pan Fluor 40× objective (N.A. 0.75), fit with appropriate filters and images captured with an Orca II CCD camera, model C4742–95 (Hamamatsu; Middlesex, NJ) and Metamorph image analysis and acquisition software (Molecular Devices; Sunnyvale, CA, USA). Images were exported to Photoshop (Adobe; San Jose, CA) and only linear adjustments to brightness and/or contrast were performed.

### Morphometric and statistical analysis

Captured images of cells containing cilia, verified by costaining with anti-acetlyated tubulin and anti-gamma tubulin for the centrosome (Thermo Scientific, PA5–34815; Asheville, NC) were captured by Metamorph and enlarged to visualize cilia clearly. The length of cilia was obtained using the line tool calibrated for the 100X objective. For each treatment, 300 cells were measured. The data for cilia quantification and length are expressed as box and whiskers plots for Figs. 1-2, 4-5 and 10 and as mean ± SEM for Figs. 6 and 7. The differences between groups were analyzed using the unpaired Student’s t-test. A *p*-value of ≤0.05 was considered significant.

### Coimmunoprecipitation

Protein complexes were collected by immunoprecipitation. Briefly, affinity purified antibody to PPP1R42 was incubated with precleared cell lysate (1 mg protein) followed by anti-rabbit IgG beads. After transfer to membrane, immunoprecipated proteins were detected with anti-Nek2 (1:500; sc-33,167; Santa Cruz Biotechnology; Dallas, TX) or anti-AurA antibodies (1:500; PC742; EMD Millipore, Billerica, MA), and Veriblot anti-rabbit HRP (Abcam; Cambridge, MA). Use of the Veriblot secondary prevents detection of IgG heavy chain. Negative control for coimmunoprecipitation was precleared lysate incubated with no antibody.

### Kinase assay

Kinase assays were conducted in kinase buffer (5 mM MOPS, pH 7.2, 2.5 mM β-glycerophosphate, 1 mM EGTA, 0.4 mM EDTA, 5 mM MgCl_2_, 0.05 mM DTT) with 1 μg myelin basic protein as substrate and 5 μCi/μl [^32^P] ATP. 50 nM Nek2 (Thermo Fisher; Waltham, MA) was incubated with varying concentrations of recombinant PPP1R42 (Biomatik; Wilmington, DE). After the designated time, reactions were terminated by spotting onto phosphocellulose P82 paper, washed extensively with 1% phosphoric acid, and the trapped radioactivity measured by scintillation counting.

### Phosphatase assay

Inhibition or activation of PP1 by R42 and R2 was accomplished using the fluorescence based RediPlate96© enzcheck serine/threonine phosphatase assay kit from Fisher Scientific (Pittsburgh, PA). Appropriate amounts of recombinant R2 or R42 (Biomatik; Wilmington, DE) were mixed in reaction buffer containing 2 mM DTT and 200 μM MnCl_2_ and added to wells containing the fluorescent phosphatase substrate. After incubation at 30 °C for 30 min, fluorescence measured at excitation/emission 358/452 nm.

## Additional files

Additional file 1: Figure S1. Protein expression in transfected cells. In all cases cell lysates were prepared from transfected cells that were grown for 24 h, serum starved for 48 h followed by reintroduction of serum for 24 h. Proteins were resolved by SDS-PAGE, transferred to membrane and probed with the indicated antibodies. (A) Cells were transfected with the indicated expression plasmids or combinations and proteins probed with anti-FLAG (R42) or anti-myc (AurA and Nek2). The AurA and Nek2 plasmids were verified by sequencing, we were unable to resolve these proteins using this gel system; however, both AurA and Nek2 have been reported as doublets in PAGE [13, 49]. Untransfected cells served as negative control (−C). (B) Cells were transfected with the indicated expression plasmids or combinations and proteins probed with anti-myc (AurA and Nek2). Untransfected cells served as negative control (−C). All panels for each section were exposed to film for the same length of time. (C) Cells were treated with off target (OT) or PPP1R42 (R42) targeting siRNA (KD) and membrane probed with anti-R42 and anti-actin. (D) Cells were transfected with R42-FLAG tagged vector or empty vector (−C) and proteins probed with anti-FLAG. Expressed proteins are maintained in the cell throughout the course of the experiment with reduction when cells are metabolically inactive after starvation. Blots were probed for actin as a loading control. (TIFF 5922 kb)

Additional file 2: Figure S2. Expression plasmids transfect ARPE-19 at high efficiency. ARPE-19 cells were transfected with plasmids expressing either FLAG tagged R42 or myc tagged kinase constructs and grown for 24 h in complete media. Cells were stained with anti-FLAG or anti-myc antibody and detected with Alex Fluor 594 secondary antibody (red). Nuclei were stained with DAPI (blue) (A). 100 cells were counted for each condition and the efficiency of transfection for all constructs was about 90%. Scale bars equal 10 μm. (B) Proteins lysates from cells transfected with either Nek2, AurA, Nek2KD, AurAKD, and R42, were separated by SDS-PAGE transferred to membrane and probed with the appropriate antibodies. Proteins from untransfected cells were loaded to indicate the level of endogenous protein (Ne, Ae, and R42e). (TIFF 16425 kb)

Additional file 3: Figure S3. PPP1R42 does not enhance PP1 activity in vitro. Recombinant PP1 (USBiologicals; Salem, MA) was incubated with varying concentrations of recombinant R2 (A) or R42 (B) (Biomatik; Wilmington, DE) and phosphatase activity measured as described in Materials and Methods. (TIFF 2235 kb)

## Abbreviations

AurA KD: Aurora A kinase dead
AurA: Aurora A
KD: kinase dead, knock down
Nek2 KD: Never in mitosis kinase-2 kinase dead
Nek2: Never in mitosis kinase-2
OT: Off target
PPP1R2 or R2: Phosphoprotein phosphatase 1 regulatory subunit 2 or Inhibitor-2
PPP1R42 or R42: Phosphoprotein phosphatase 1 regulatory subunit 42
