# Supplementary figures and images for: A novel interaction between kinase activities in regulation of cilia formation
Source: BMC Cell Biol. 2017 Nov 15;18:33. doi: 10.1186/s12860-017-0149-5 (PMC5688660; doi:10.1186/s12860-017-0149-5)

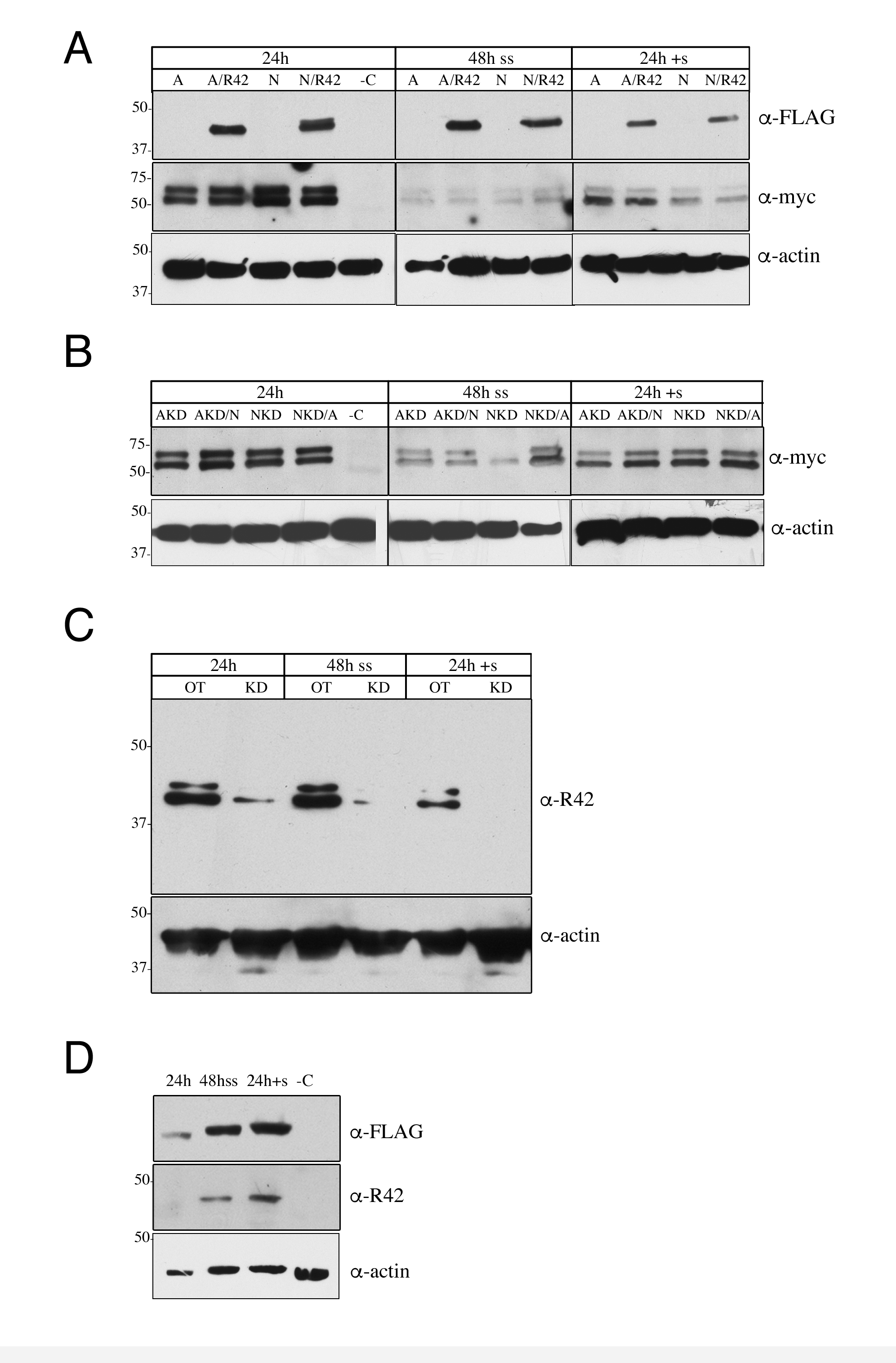

Supplement: Supplementary file 1 — Protein expression in transfected cells. In all cases cell lysates were prepared from transfected cells that were grown for 24 h, serum starved for 48 h followed by reintroduction of serum for 24 h. Proteins were resolved by SDS-PAGE, transferred to membrane and probed with the indicated antibodies. (A) Cells were transfected with the indicated expression plasmids or combinations and proteins probed with anti-FLAG (R42) or anti-myc (AurA and Nek2). The AurA and Nek2 plasmids were verified by sequencing, we were unable to resolve these proteins using this gel system; however, both AurA and Nek2 have been reported as doublets in PAGE [13, 49]. Untransfected cells served as negative control (−C). (B) Cells were transfected with the indicated expression plasmids or combinations and proteins probed with anti-myc (AurA and Nek2). Untransfected cells served as negative control (−C). All panels for each section were exposed to film for the same length of time. (C) Cells were treated with off target (OT) or PPP1R42 (R42) targeting siRNA (KD) and membrane probed with anti-R42 and anti-actin. (D) Cells were transfected with R42-FLAG tagged vector or empty vector (−C) and proteins probed with anti-FLAG. Expressed proteins are maintained in the cell throughout the course of the experiment with reduction when cells are metabolically inactive after starvation. Blots were probed for actin as a loading control. (TIFF 5922 kb) [file 12860_2017_149_MOESM1_ESM.tif]

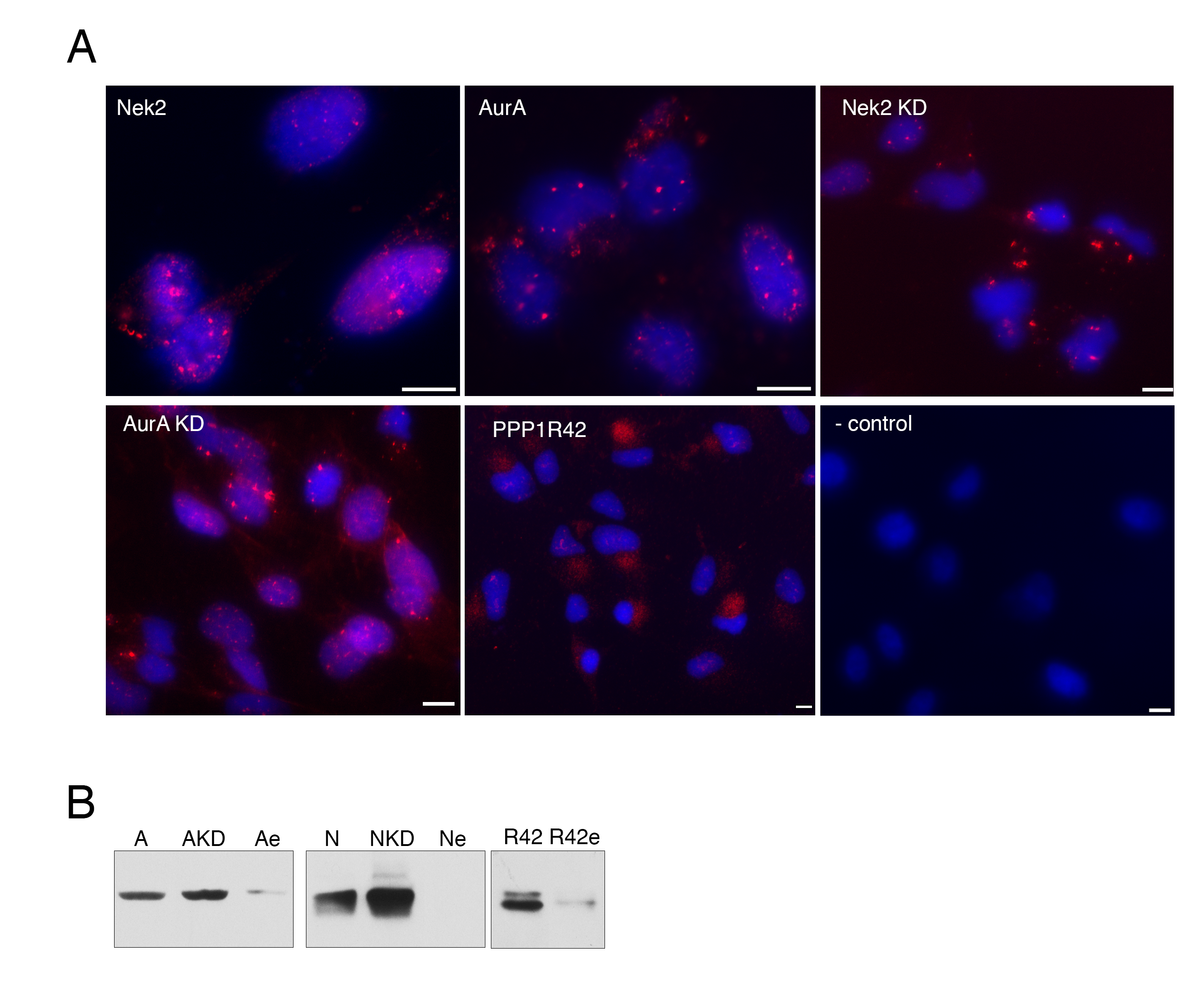

Supplement: Supplementary file 2 — Expression plasmids transfect ARPE-19 at high efficiency. ARPE-19 cells were transfected with plasmids expressing either FLAG tagged R42 or myc tagged kinase constructs and grown for 24 h in complete media. Cells were stained with anti-FLAG or anti-myc antibody and detected with Alex Fluor 594 secondary antibody (red). Nuclei were stained with DAPI (blue) (A). 100 cells were counted for each condition and the efficiency of transfection for all constructs was about 90%. Scale bars equal 10 μm. (B) Proteins lysates from cells transfected with either Nek2, AurA, Nek2KD, AurAKD, and R42, were separated by SDS-PAGE transferred to membrane and probed with the appropriate antibodies. Proteins from untransfected cells were loaded to indicate the level of endogenous protein (Ne, Ae, and R42e). (TIFF 16425 kb) [file 12860_2017_149_MOESM2_ESM.tif]

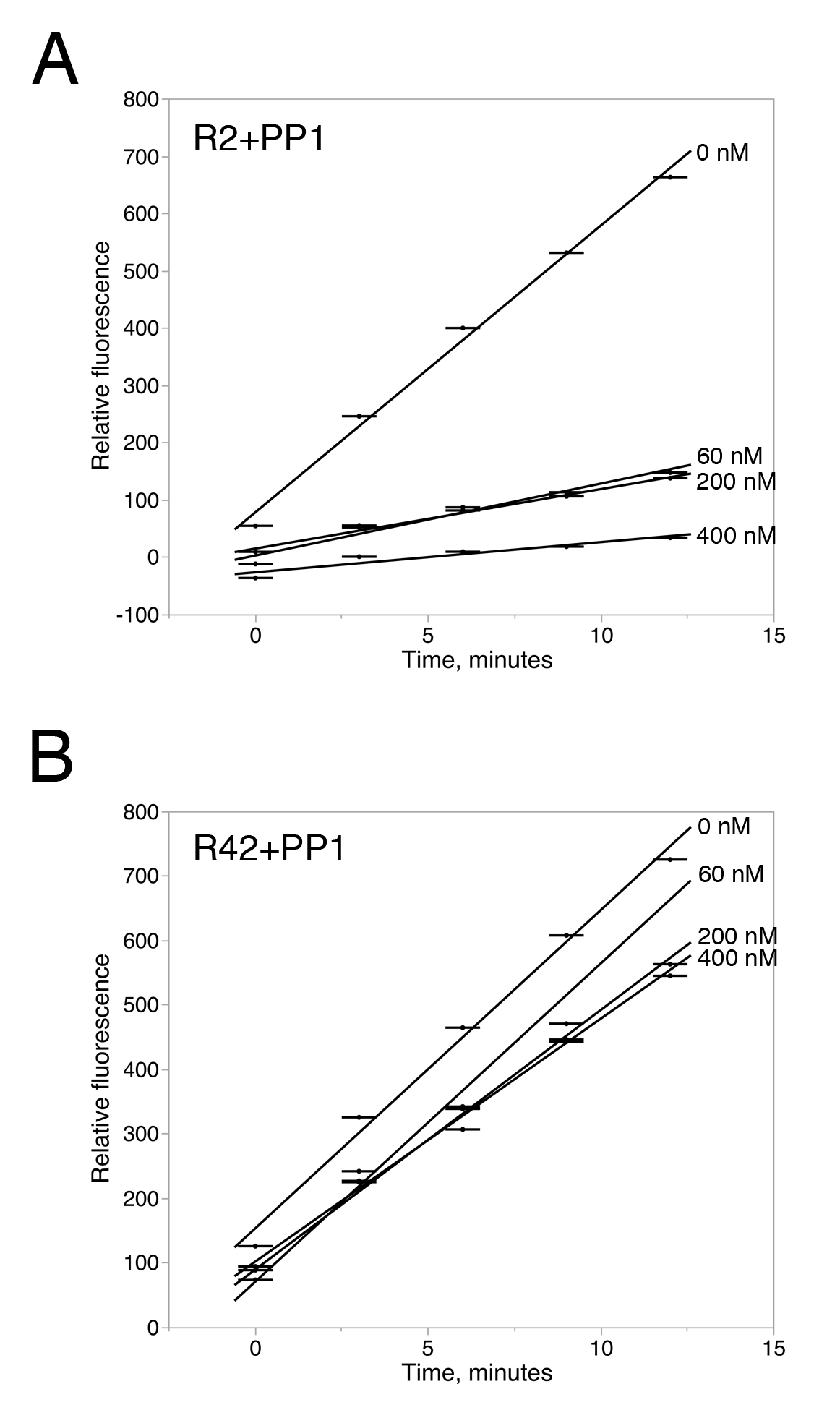

Supplement: Supplementary file 3 — PPP1R42 does not enhance PP1 activity in vitro. Recombinant PP1 (USBiologicals; Salem, MA) was incubated with varying concentrations of recombinant R2 (A) or R42 (B) (Biomatik; Wilmington, DE) and phosphatase activity measured as described in Materials and Methods. (TIFF 2235 kb) [file 12860_2017_149_MOESM3_ESM.tif]
